# Environmental exposure, airway microbiome and respiratory health: You are what you breathe

**DOI:** 10.1002/ctm2.1394

**Published:** 2023-08-30

**Authors:** Xinzhu Yi, Hanqin Cai, Jingyuan Gao, Zhang Wang

**Affiliations:** ^1^ Institute of Ecological Sciences, School of Life Sciences South China Normal University Guangzhou China

## 1 INTRODUCTION TO THE STUDY

**FIGURE 1:**
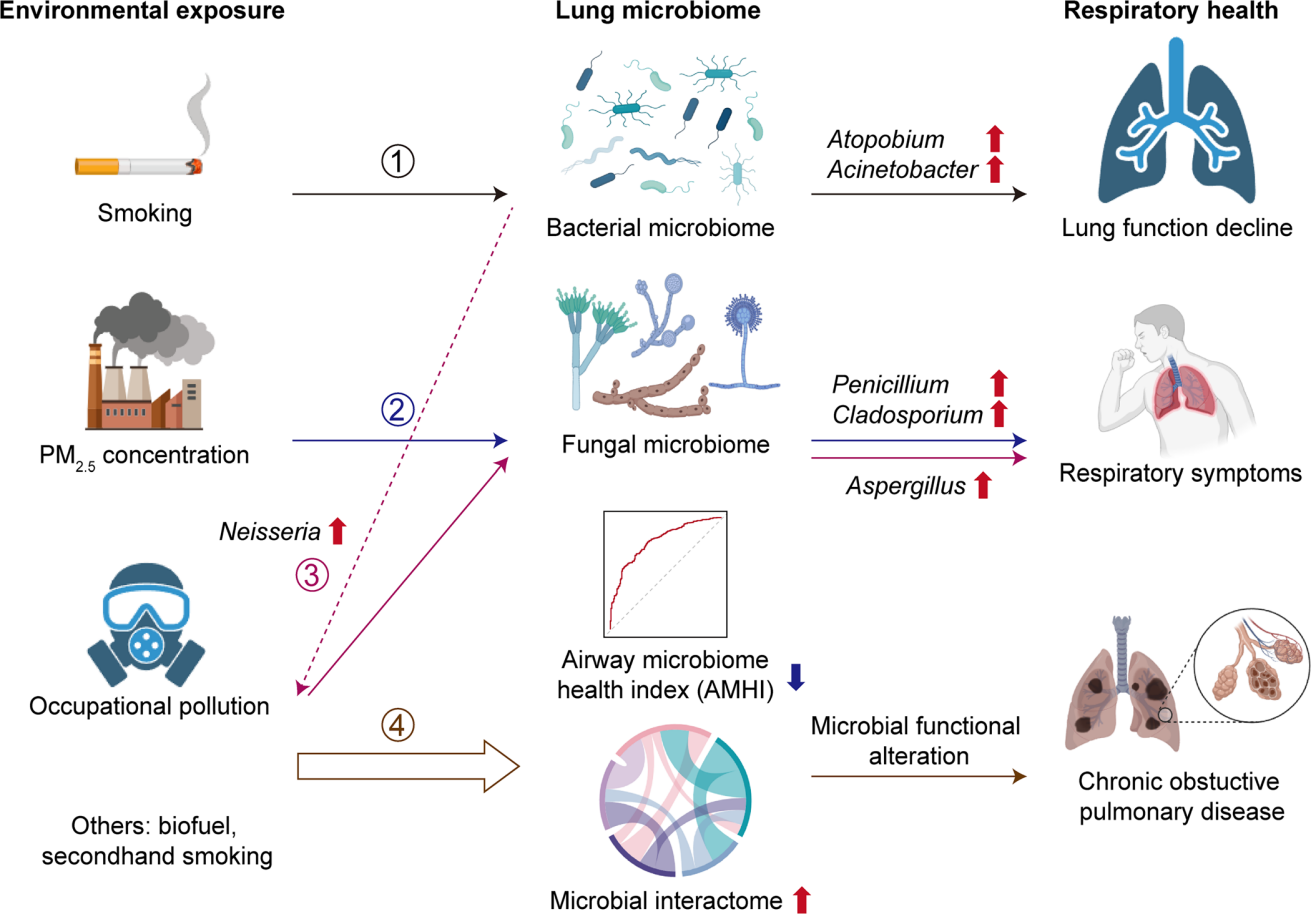
An overview of the findings on the role of the airway microbiome in mediating environmental exposure and respiratory health. Highlighted are the four major findings of the study, where (1) bacterial microbiome mediates the effects of smoking on lung function decline; (2) fungal microbiome mediates the effects of PM_2.5_ concentration on respiratory symptoms; (3) enrichment of *Neisseria* (in dashed line) associates with a greater influence of occupational pollution on respiratory symptoms; (4) exposure associates with a declined airway microbiome health index and an enhanced interactome, resembling those seen in chronic obstructive pulmonary disease.

Our human respiratory system is constantly exposed to the stimuli of environmental toxicants, which is known to trigger a dysregulated lung immune response and lead to respiratory symptoms, impaired lung function and development of respiratory diseases.^1^, ^2^ Mounting evidence suggests the existence of a microbial community or microbiome, composing of bacteria, fungi, and viruses, in healthy human lungs, which plays an indispensable role in respiratory health and diseases.^3^, ^4^ Nevertheless, it remains unclear how the airway microbial ecosystem responds to environmental exposure and whether it is implicated in the exposure's effects on respiratory health. Through a population‐based microbiome survey in Guangdong province, China, we have recently showed that the airway microbiome not only mediates the effects of environmental exposure on respiratory health, but also may impact an individual's susceptibility to exposure (Figure 1), thereby could be a promising new source of biomarkers and targets for environmental risk evaluation and prevention.^5^

## 2 THE POPULATION‐BASED AIRWAY MICROBIOME SURVEY

Despite extensive studies on the airway microbiome in patients with respiratory diseases, characterization of the airway microbiome in healthy populations remains largely scarce, partly due to the unique challenges in obtaining lower airway specimens from healthy individuals. Leveraging a province‐wide chronic obstructive pulmonary disease (COPD) surveillance by Guangdong Provincial Center for Disease Control and Prevention, we have surveyed a total of 3915 household members from six districts, 18 sub‐districts, and 36 neighborhoods in Guangdong, China. Induced sputum was collected and the microbiome (including bacterial, fungal taxa and metagenomes) was characterized in 1651 participants. In terms of exposure, smoking, biofuel, occupational pollution, PM_2.5_ concentration, and secondhand smoking were assessed. In terms of respiratory health conditions, lung function was measured by spirometry, and information regarding respiratory symptoms (cough, dyspnea, phlegm, wheeze, COPD Assessment Test [CAT] score) was collected.

## 3 EXPOSURE INFLUENCES THE RESPIRATORY HEALTH THROUGH THE AIRWAY MICROBIOME

A bi‐directional mediation analysis revealed a mediation role of the airway microbiome between exposure and respiratory health, where distinct entities of the microbiota were found to mediate the effects of different exposures. Specifically, bacterial microbiota were found to mediate the influence of smoking on the lung function, where *Acinetobacter* was found in particular to drive the mediation of microbial functional attributes. On the other hand, fungal microbiota, such as *Penicillium* and *Cladosporium*, were found to mediate the influence of PM_2.5_ concentration on respiratory symptom (CAT score). Through further statistical modeling, bacterial microbiota, and specifically *Neisseria*, a potential airway pathobiont,^6^ was found to exhibit a significant interaction effect with occupational pollution, in relation to the CAT score. In particular, individuals with a higher abundance of *Neisseria* in their airways experienced a greater respiratory symptom burden in response to occupational pollution. This is accompanied with unique changes in the fungal microbiota in particular an increase in *Aspergillus*, the commonly observed fungi in the environment.^7^ Therefore, the airway microbiome is not only altered by exposure but may in turn modulate its effects on respiratory health.

## 4 A MICROBIOME‐BASED QUANTITATIVE FRAMEWORK FOR EXPOSURE AND RESPIRATORY HEALTH

A signature for healthy airway microbiome could serve as a baseline to assess dysbiosis in respiratory diseases. To this end, an airway microbiome health index (AMHI) was developed, by simultaneously integrating bacterial and fungal taxa to assess an individual's respiratory health status. AMHI was declined in airway diseases compared with healthy individuals, declined in individuals with respiratory symptom, and continuously declined in individuals experiencing accumulated exposures. AMHI further interacts with exposure on its effect on respiratory health, together suggesting the potential utility of this microbiome‐based scoring system in assessing an individual's respiratory healthy status and susceptibility to exposure. A continuous decline of AMHI was observed from healthy individuals, individuals ‘at‐risk’ for COPD (pre‐COPD), to those diagnosed with COPD. In concert, there was a continuous expansion in the microbial interaction network from healthy, pre‐COPD to COPD. Among all exposures, smoking was associated with the greatest network perturbation. Microbiome taxa that drive the network perturbation were found to contribute to functional shifts from healthy, pre‐COPD and COPD, suggesting the interactome changes could be implicated in early development of COPD. AMHI and the interactome have the potential to assist in quantifying the individualized effects of exposure on respiratory health and early development of COPD.

## 5 IMPLICATION FROM CURRENT FINDINGS AND FUTURE OUTLOOK

Collectively, we showed an airway microbial ecosystem that can both be influenced by exposure and in turn modulate its impact on the respiratory health. Together, they highlight the central role of the airway microbiome in shaping an individual's respiratory health and response to environmental exposure (you are what you breathe). The implications of these results are several. First, it may be possible to assess an individual's susceptibility to exposure based on the composition of the airway microbiome. For instance, individuals with enrichment of *Neisseria* in the airways could be more vulnerable to the effects of the occupational pollution on the development of respiratory symptoms. Identifying this subgroup of population is important, as preventative measures can be taken to limit exposure for these individuals. The formulation of AMHI may further allow for a personalized scoring system to assess an individual's risk of developing chronic airway diseases as a result of exposure. Second, in light of the recently renewed interest on early‐stage COPD,^8^ the airway microbiome, in particular AMHI and the interactome, could serve as potential novel markers to indicate early COPD development. Third, given the pivotal role of the airway microbiome in mediating the effect of exposure on respiratory health, it is of utmost interest to explore possible airway microbiome modulation strategies to prevent the risk of respiratory health impairment caused by exposure. Future studies are warranted to assess the variation of the airway microbiome across populations with broader geographic variations, and with different genetic backgrounds, socioeconomic status, and exposure history, to identify the specific microbes, the effector molecules they produce (i.e., metabolites, proteins), and the host genes and pathways involved in mediating exposure's effect on respiratory health using a multi‐omic approach,^9^, ^10^ and to elucidate the biological mechanisms underlying such environment‐microbiome‐host interplay. These efforts are essential towards a possible real‐world application of the airway microbiome in assisting in environmental risk assessment, stratification and prevention.

## CONFLICT OF INTEREST STATEMENT

The authors declare no conflict of interest.
